# Disturb mitochondrial associated proteostasis: Neurodegeneration and imperfect ageing

**DOI:** 10.3389/fcell.2023.1146564

**Published:** 2023-03-10

**Authors:** Yuvraj Anandrao Jagtap, Prashant Kumar, Sumit Kinger, Ankur Rakesh Dubey, Akash Choudhary, Ravi Kumar Gutti, Sarika Singh, Hem Chandra Jha, Krishna Mohan Poluri, Amit Mishra

**Affiliations:** ^1^ Cellular and Molecular Neurobiology Unit, Indian Institute of Technology Jodhpur, Jodhpur, Rajasthan, India; ^2^ Department of Biochemistry, School of Life Sciences, University of Hyderabad, Hyderabad, India; ^3^ Division of Neuroscience and Ageing Biology, Division of Toxicology and Experimental Medicine, CSIR-Central Drug Research Institute, Lucknow, India; ^4^ Infection Bioengineering Group, Department of Biosciences and Biomedical Engineering, Indian Institute of Technology Indore, Indore, Simrol, India; ^5^ Department of Biotechnology, Indian Institute of Technology Roorkee, Centre for Nanotechnology, Indian Institute of Technology Roorkee, Roorkee, Uttarakhand, India

**Keywords:** neurodegeneration, mitostasis, autophagy, proteasome, oxidative stress, mitochondria

## Abstract

The disturbance in mitochondrial functions and homeostasis are the major features of neuron degenerative conditions, like Parkinson’s disease, Amyotrophic Lateral Sclerosis, and Alzheimer’s disease, along with protein misfolding. The aberrantly folded proteins are known to link with impaired mitochondrial pathways, further contributing to disease pathogenesis. Despite their central significance, the implications of mitochondrial homeostasis disruption on other organelles and cellular processes remain insufficiently explored. Here, we have reviewed the dysfunction in mitochondrial physiology, under neuron degenerating conditions. The disease misfolded proteins impact quality control mechanisms of mitochondria, such as fission, fusion, mitophagy, and proteasomal clearance, to the detriment of neuron. The adversely affected mitochondrial functional roles, like oxidative phosphorylation, calcium homeostasis, and biomolecule synthesis as well as its axes and contacts with endoplasmic reticulum and lysosomes are also discussed. Mitochondria sense and respond to multiple cytotoxic stress to make cell adapt and survive, though chronic dysfunction leads to cell death. Mitochondria and their proteins can be candidates for biomarkers and therapeutic targets. Investigation of internetworking between mitochondria and neurodegeneration proteins can enhance our holistic understanding of such conditions and help in designing more targeted therapies.

## 1 Introduction

The cellular system continuously performs multiple biochemical reactions to maintain its homeostasis essential for sustaining life. These biochemical reactions need energy generated by the organelle mitochondria, also recognized as “cellular powerhouse.” In 1898, Carl Benda coined the term mitochondria for the intracellular structures noted by Richard Altman (1890) that are ubiquitously found in eukaryotes (Ernster and Schatz, 1981). Altman showed the involvement of these structures in cellular metabolism and suggested its existence to be symbiotic. Lynn Margulis further laid down the basis for the endosymbiotic theory, where she proposed the hypothesis; that engulfment of prokaryotes (capable of aerobic metabolism) by heterotrophic anaerobe for survival in the oxygen-rich environment (Sagan, 1967). Although numerous reports support this theory, several other theories were also suggested for origin of mitochondria (Gray, 2012; Martin et al., 2015; Dunn, 2017). The mitochondria are essential for cellular energy and perform numerous other functions equally vital for normal cell functioning. The altered functioning of mitochondria is reported in several pathological conditions, including metabolic, neurodevelopmental, neurodegeneration, and cancer (Vyas et al., 2016; Srivastava, 2017; Ortiz-Gonzalez, 2021). Here in this review, we have focused on how mitochondrial damage and dysfunction are linked with multiple neurodegenerative diseases. Considering mitochondria’s complex and essential functions in diverse cellular pathways and signaling, maintaining its health and dynamics is critical. Several quality control pathways are engaged in mitochondrial homeostasis and its proper functioning during stress conditions. Various neurodegenerative disease-causing proteins are observed to alter this homeostasis by interfering with the mitochondrial pathways affecting their normal functioning (Briston and Hicks, 2018). Furthermore, how these mitochondrial deformities lead to other cellular defects is also reviewed. Identifying the multiple mitochondria-associated disease targets and pathways can help in detailed understanding of disease pathology and developing more specified therapeutic options.

### 1.1 Structural and functional importance of mitochondria

**FIGURE 1:**
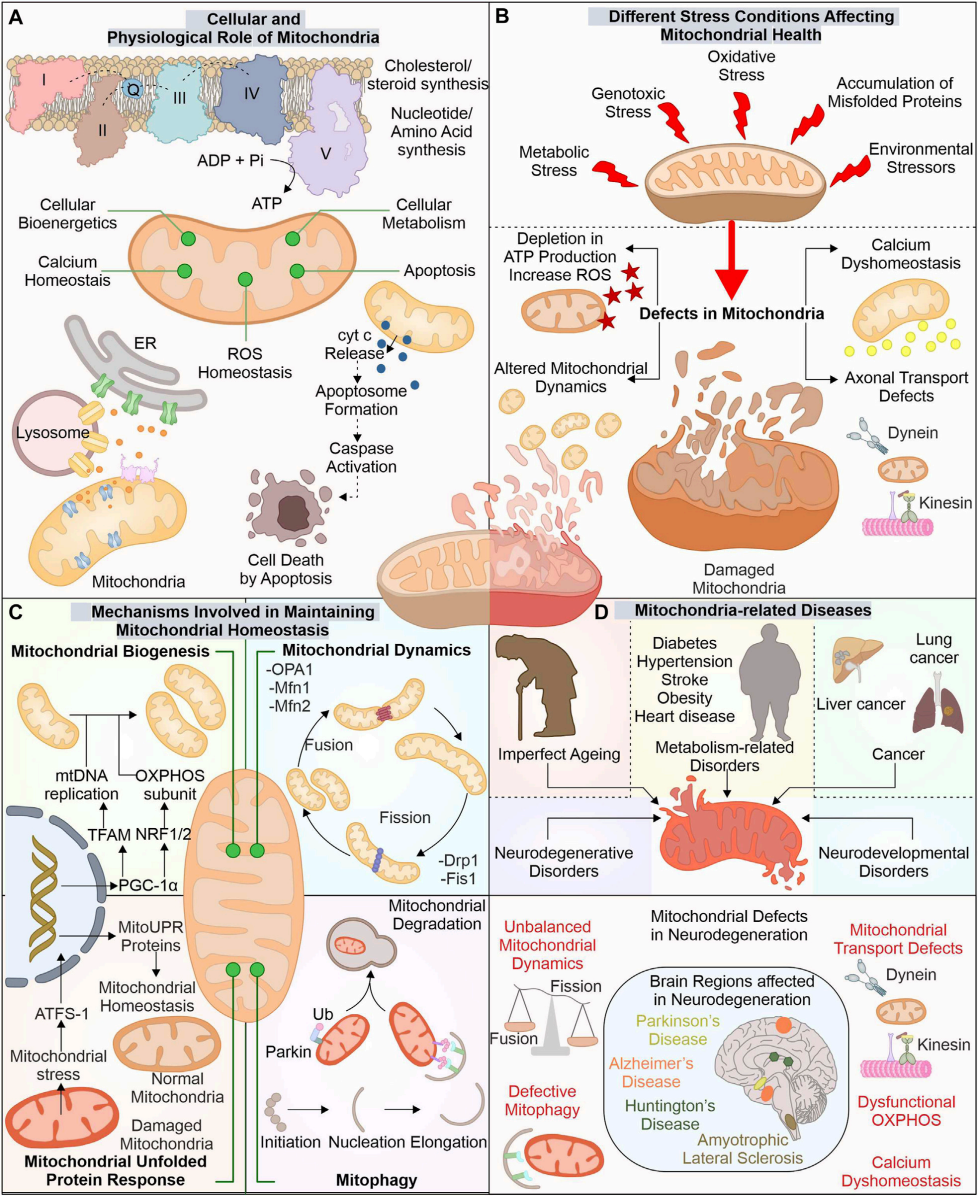
Mitochondria: The key player in cellular function and its implication in diverse diseases. Besides producing ATP for numerous cellular pathways, mitochondria also regulate ROS and calcium levels, synthesize several biomolecules, and control cell death *via* the apoptosis pathway **(A)**. Different stresses affect these mitochondrial functions leading to mitochondrial dysfunction **(B)**. Mitochondria possess several pathways to avoid such damage by controlling the synthesis of new mitochondria, morphology, and eliminating any dysfunctional mitochondria **(C)**. The altered function of these mitostasis mechanisms is observed in several disease conditions, including neuronal degenerative disorders such as ALS, AD, PD, and HD **(D)**.

The advancement in electron microscopy has enhanced our structural understanding of mitochondria. The mitochondria exist with outer mitochondrial membrane (OMM) isolating it from cytoplasm. Inner mitochondrial membrane (IMM) folded in cristae-like structures provides a site for complexes of electron transport chain (ETC.) (ATP synthesis). The space between these two membranes, i.e., intermembrane space (IMS), contains cytochrome c (*Cyt C*), essential for induction of the cellular death pathway *via* apoptosis (Wang and Youle, 2009). Mitochondria perform numerous functions, like regulation of cytosolic calcium levels and formation of biomolecules (cholesterol, steroid, and heme), and generate reactive oxygen species (ROS) (Lathrop and Timko, 1993; Gunter et al., 1994; Miller, 2013) (Figure 1).

The OMM tethers with different organelles with the help of numerous membrane-localized proteins. Mitochondria interact with endoplasmic reticulum (ER) membrane *via* mitochondrial-associated ER membrane (MAM) proteins; its altered association is reported in diseases like neurodegeneration (Lee and Min, 2018). The IMM is divided into two separate components, i.e., inner boundary membrane (IBM), present close to OMM, and the cristae membrane (CM), having larger section folded into the mitochondrial matrix, with cristae junctions connecting these two parts (Frey et al., 2002). The protein composition in these separate membranes differs as CM has a high number of enzymes involved in OXPHOS (Vogel et al., 2006). The different cellular stress factors can damage mitochondrial health leading to its altered functions with depletion in ATP production and induced ROS levels generating excessive oxidative damage. These stress factors can include exposure to ultraviolet (UV) light, excessive glucose levels, genotoxic stress, and H_2_O_2_ production, which affect the morphology of mitochondria (Tondera et al., 2009; Trudeau et al., 2011; Iqbal and Hood, 2014).

### 1.2 Mechanisms involved in maintaining mitochondrial homeostasis

The cell has developed different mechanisms and pathways to survive and adapt during stressful conditions, including mitochondrial quality control mechanisms. Which modulate the mitochondrial functions and their number as per the cellular requirements. The biogenesis of new mitochondria is does not occur through a *de novo* pathway, but is divided from the existing one. The formation of new mitochondria needs replication of mitochondria DNA (mtDNA) and expression of multiple mitochondrial proteins. Nearly 1,136 genes are known till now with expressing proteins of mitochondrial origin. The mitochondrial protein synthesis is controlled by nuclear genome (∼99%) and mtDNA (13 proteins) (Scarpulla, 2008; Rath et al., 2021). The biogenesis of mitochondria is a highly controlled mechanism requiring concerted expression of mtDNA and nucleus DNA. PGC-1 acts as a master controller for biogenesis of mitochondria by modulating the transcription factors (TF) required for mtDNA copying and its gene expression (Wu et al., 1999). The PGC-1 levels are controlled by different cellular signals, such as cellular energy and metabolism, where AMPK and SIRT1 act as modulators of PGC-1 activity (Scarpulla, 2011). The targets of PGC-1 include ERRα, NRF1, and NRF2, which modulate mitochondrial biogenesis gene expression. NRF1/2 controls mitochondrial respiratory chain protein synthesis, membrane protein import complexes, and other TFs (TFAM, TFB1M, and TFB2M) (Bruni et al., 2010; Satoh et al., 2013; Yang et al., 2014). TFAM maintains number of mtDNA and is crucial in biogenesis as its inactivity alters mtDNA copies and OXPHOS (Larsson et al., 1998). The depletion of PGC-1α activity is reported in aging and associated disorders (Sahin and DePinho, 2012; Picca et al., 2013).

Furthermore, fission and fusion pathways regulate mitochondrial dynamics under changing metabolic requirements of cells. As during metabolic and environmental stress, the damaged mitochondria are fused with healthy ones to reduce the damage. Moreover, through the mitochondrial fission mechanism, the damaged mitochondrial part is removed (Youle and van der Bliek, 2012; van der Bliek et al., 2013). Drp1 (Dnm1 in yeast) protein is involved in fission pathway (Bleazard et al., 1999; Smirnova et al., 2001). Fis1 protein aids in the recruitment and proper distribution of Drp1 at the site of mitochondrial division. Further, Drp1, through its GTPase activity, helps in membrane constriction and, finally, division of organelle (Mozdy et al., 2000; Ingerman et al., 2005). The OMM and IMM fusion is controlled through separate set of proteins. Mfn1 and Mfn2 recruited at the OMM interact with adjoining mitochondria-localized mitofusion proteins. Further, their GTPase activity helps in the fusion between these two separate mitochondrial membranes (Santel and Fuller, 2001). The IMM fusion is controlled by another GTPase protein, OPA1, localized at the IMM protruding into IMS (Olichon et al., 2002). OMA1, an IMM localized protease, can decrease the OPA1 activity and prevent the fusion of damaged mitochondria with decreased membrane potential (ΔΨm) and ATP levels (Head et al., 2009; Youle and van der Bliek, 2012).

Mitochondrial chaperones and proteases are also involved in maintaining mitochondrial health by either refolding or removing unfolded proteins and mediating their degradation. Numerous such chaperones are distributed in mitochondria performing specified functions. At OMM, Hsp90, together with TOM, assists in the import of mitochondrial proteins. Further, HtrA2 (at IMS) helps translocate proteins from outer to inner mitochondrial membrane. At IMM, chaperones such as COX17p control the folding of, ETC., complex proteins. Finally, at the mitochondrial matrix, different chaperones and co-chaperones (Hsp60-Hsp10, mtHsp70-HSC20, DNAJA3, ERAL1) regulate the maintenance of matrix proteins, mtDNA, and mitoribosome (Castro et al., 2018). Furthermore, proteases like ClpP and LonP (Matrix localized), m-AAA and i-AAA proteases (IMM), and HtrA2 (IMS), target degradation of misfolded and damaged proteins. Furthermore, these proteases also regulate fusion and fission by modulating proteins involved in these pathways (de Sagarra et al., 1999; Leonhard et al., 2000; Bota and Davies, 2002; Martins et al., 2004). The OMM localized unfolded proteins are removed through assistance from cytosolic proteasome system (Livnat-Levanon and Glickman, 2011).

However, with the increased accumulation of unfolded proteins and oxidative stress, mitochondria activate retrograde signaling, also called mitochondrial unfolded protein response (UPR^mt^) (Haynes et al., 2007; Kohler et al., 2015; Oliveira and Hood, 2018). The ectopic expression of non-native ornithine transcarbamylase in COS-7 cells causes its accumulation in mitochondria (Zhao et al., 2002). This mitochondrial accumulation of misfolded protein was found to induce different genes, including chaperones (Hsp60, mtDnaJ, Hsp10), proteases (YMEL1 and ClpP) along with complex I subunit and membrane-localized Tim17A import protein (Zhao et al., 2002; Aldridge et al., 2007). HAF-1 represses the import of ATFS-1 (ATF5 in humans) transcription factor in defective mitochondria leading to its translocation to the nucleus (Fiorese et al., 2016; Shpilka and Haynes, 2018). ATFS-1 with DVE-1 and UBL-5 remodulate chromatin structure, inducing mitochondrial related gene transcripts production such as chaperones (Hsp60, Dnj-10) and antioxidants gene transcription factor (Skn-1) (Nargund et al., 2012; Shpilka and Haynes, 2018). Mitophagy is another quality control pathway where selective removal of altered mitochondria occurs. PINK1 and E3 ubiquitin ligase Parkin are crucial components of mitophagy. The proteolytic cleavage inhibition of PINK1 in mitochondria with reduced membrane potential causes its stabilization on the mitochondrial surface (Matsuda et al., 2010; Narendra et al., 2010; Fallaize et al., 2015). Furthermore, the stabilized PINK1 phosphorylates and recruits Parkin to mitochondria, inducing its ubiquitination activity (Kim et al., 2008; Narendra et al., 2008; Matsuda et al., 2010; Sha et al., 2010). The PINK1-recruited Parkin initiates the ubiquitination of multiple proteins localized to OMM, targeting its degradation by ubiquitin-proteasome system (UPS). The clearance of multiple such OMM localized proteins (Mfn1, Mfn2) *via* UPS is an essential checkpoint for mitophagy (Chan et al., 2011).

### 1.3 Mitochondrial impairments in complex disorders: Focus on neurodegeneration

The cellular importance of mitochondria is well acknowledged; therefore, it is not surprising that any damage or dysfunction in this organelle leads to disease conditions (McInnes, 2013; Hsu et al., 2016; Monzio Compagnoni et al., 2020). The mitochondria-related diseases commonly occur because of mutations (spontaneous/inherited) in nuclear and mtDNA encoding proteins for mitochondrial functions such as OXPHOS complexes, mitochondrial quality control proteins, and enzymes involved in mtDNA preservation and expression of genes (Gorman et al., 2016). Mutations in mtDNA are reported in diseases such as Leigh syndrome, LHON, and MERRF (Gorman et al., 2015; Gorman et al., 2016). Further multiple metabolic disorders such as diabetes, hypertension, stroke, obesity, and heart disease were also reported with mitochondrial impairment (Bhatti et al., 2017). Earlier the Warburg effect observed in cancer cells was thought to be linked with mitochondrial dysfunction; however, later studies confirm the importance of mitochondrial energetics in survival and growth of cancer (Wallace, 2012). The augmented activity of HIF1α and FOS–JUN inducing cancer cell proliferation was identified to be associated with increased mitochondrial ROS levels. Moreover, mitochondrial calcium dyshomeostasis leads to cytosolic accumulation of calcium, further inducing the metastatic potential of cancer cells by activating NF-κB (Wallace, 2012).

The exposure to different stress conditions and impaired quality control pathways further aggravates the mitochondrial damage altering its normal functions. The neuronal cells (affected in neurodegeneration), with their complex cellular structure and postmitotic nature, depend on mitochondrial energy for numerous pathways and are the majorly affected cells due to their altered function. Mitochondrial dynamics, morphology, and function changes are reported in amyotrophic lateral sclerosis (ALS) disease models (Magrane et al., 2014; Stribl et al., 2014). Furthermore, Huntington’s disease was observed to have mitochondrial defects, altered OXPHOS, and impaired transport of mitochondrial proteins (Kim et al., 2010; Yano et al., 2014). Alzheimer’s disease patients were also reported with altered mitochondrial morphology, defective biogenesis, and reduced levels of respiratory complexes involved in, ETC (Maurer et al., 2000; Manczak et al., 2004; Baloyannis, 2006; Sheng et al., 2012). The mitochondrial quality control defects are also apparent in Parkinson’s disease (PD) patients having PINK1, DJ-1, and Parkin gene mutations (Kitada et al., 1998; Bonifati et al., 2003; Valente et al., 2004). Also, mitochondrial energy defects with deficient capacity of complex I are reported in PD (Gatt et al., 2016).

## 2 Deciphering the link between amyotrophic lateral sclerosis and mitochondrial impairment

**FIGURE 2:**
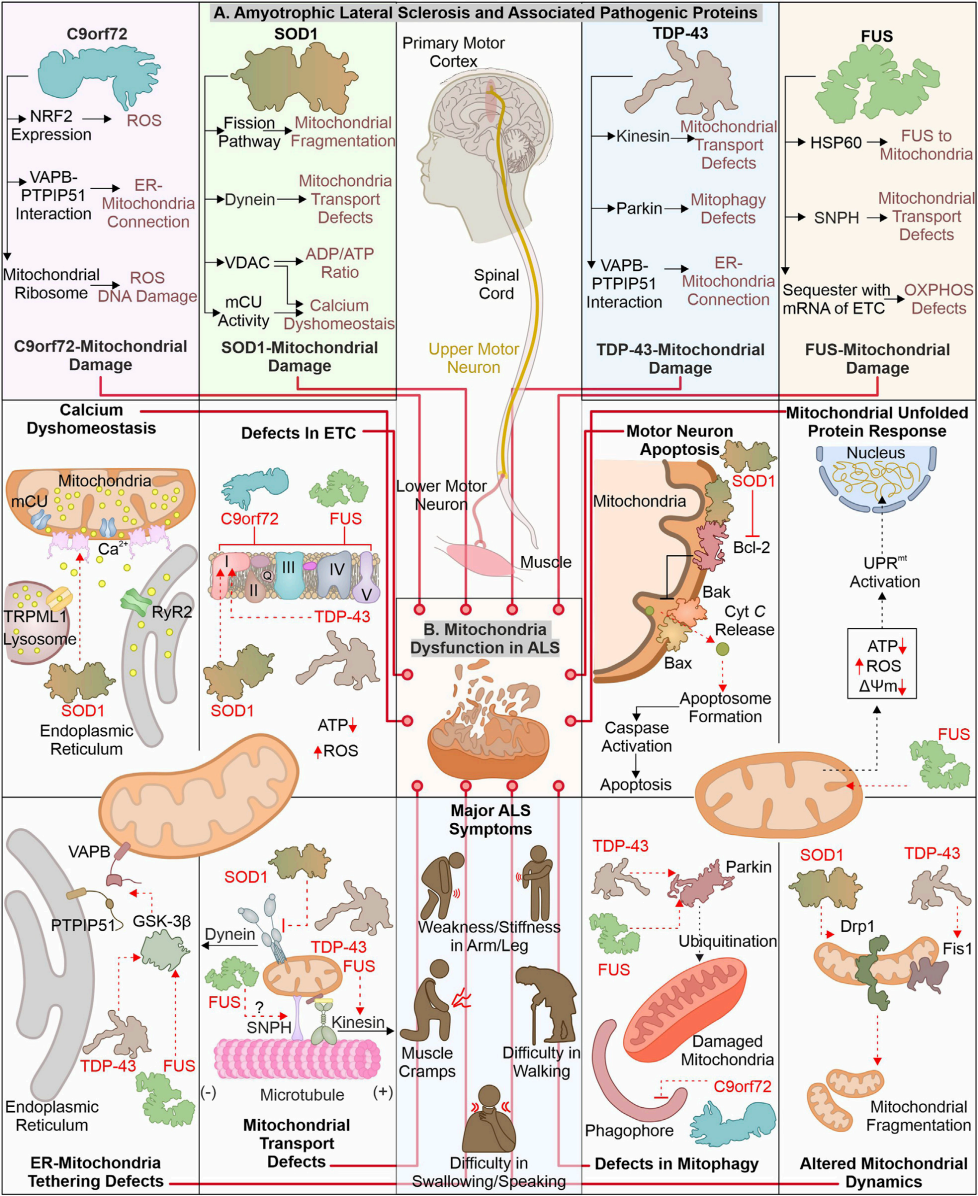
ALS-linked mutant proteins contribute to mitochondrial impairment. ALS is a complex neuronal degenerative condition where motor neurons (brain and spinal cord) are affected. Various signs, like muscle cramps, stiffness in the arm, and swallowing and speaking difficulties, characterize ALS patients. Different disease-causing proteins are identified to interact and affect mitochondrial proteins causing mitochondrial damage at several levels **(A).** The interaction of ALS-linked pathogenic proteins (TDP-43, SOD1, C9orf72, and FUS) with mitochondria and associated proteins causes its dysfunction, leading to calcium dyshomeostasis, altered OXPHOS, mitochondrial dynamics, and mitophagy **(B)**.

ALS is identified with motor neuronal deterioration in brain and spinal cord region that regulate muscle activity. ALS is also recognized by the name “Charcot’s disease” for the contribution made by Jean-Martin Charcot to understanding ALS pathology (Goetz, 2000). ALS patients can be characterized based on symptoms such as limb onset ALS, where muscle cramps, weakness, or stiffness in the arm/leg are observed. In contrast, patients with bulbar onset ALS have swallowing and speaking difficulties, which are associated with atrophy of the tongue (Goetz, 2000; Hardiman et al., 2017). During pathogenesis of ALS, the initial muscle damage further causes paralysis of other muscles in the late stage of disease, resulting in death often due to respiratory insufficiency (Corcia et al., 2008). Numerous ALS (familial and sporadic) disease-causing mutations are identified in different genes (Renton et al., 2014; Weishaupt et al., 2016). ALS’s most frequently mutated genes include C9orf72, SOD1, TDP-43, and FUS (Figure 2). The gain or loss of function due to these mutations alters their cellular role. Furthermore, these pathogenic variants also affect cellular organelles such as mitochondria. The altered mitochondrial bioenergetics in the presence of ALS-associated pathogenic proteins also disturbs other cellular pathways generating a cytotoxic environment. Additionally, the inability of quality control pathways to maintain homeostasis by alleviating or restricting the damage caused by defective mitochondria results in cell death.

### 2.1 How are mitochondrial defects associated with the loss or gain of functions in C9orf72?

The mutations in the C9orf72 are considered as the frequently occurring genetic insult in ALS/FTD disease process. Mutations in C9orf72 ALS/FTD patients having a hexanucleotide (GGGGCC) repeats expansion (HRE) positioned at first intron of gene were reported in 2011 (DeJesus-Hernandez et al., 2011; Renton et al., 2011). The gene encoding C9orf72 is present on chromosome 9p21.2 (human) (Boxer et al., 2011). Two isoforms of C9orf72 are reported in humans, i.e., C9-S (exon 2–5, 222 amino acids) and C9-L (exon 2–11, 481 amino acids). The C9-L isoform is principally present in the brain; however, its decreased levels are reported in ALS (Frick et al., 2018). 2–30 HRE are considered normal, whereas patients with C9orf72 ALS are reported to have nearly thousands of repeats (Beck et al., 2013; Benussi et al., 2014; Hubers et al., 2014). The involvement of C9orf72 in ALS pathology remains debated as gain as well as loss of function are described to cause ALS. Multiple studies report that decreased C9orf72 at mRNA and protein levels are linked with disease pathology (Gijselinck et al., 2012; Waite et al., 2014; Saberi et al., 2018). Moreover, the higher levels of C9orf72 were found to have advantageous effects (van Blitterswijk et al., 2015). Conversely, a gain of function (at RNA or protein level) is also reported in ALS pathology. The RNA transcribed from disease-linked C9orf72 tends to form aggregates in nuclear foci (Donnelly et al., 2013; Sareen et al., 2013). Moreover, the production of different dipeptide repeats (DPRs) from expanded mRNA is also involved in ALS pathomechanism (Mori et al., 2013). Various such DPRs, including poly- (GP), (PA), (GR), (GA), and (PR), are reported in the cytoplasm of neurons (Ash et al., 2013).

The pathogenic variants of C9orf72 were found to cause mitochondrial defects where the mouse model expressing poly-(GR) form shows neuronal loss and behavioral changes. Furthermore, poly-(GR) induces ATP5A1 (subunit of complex V) proteasomal degradation reducing its levels and compromising mitochondrial function. The reduction in poly-(GR) or increased expression of ATP5A1 was found to have protective effects by reducing the neurotoxicity caused due to poly-(GR) (Choi et al., 2019). The RNAseq analysis of ALS patient tissue samples shows a decreased expression of mtDNA-encoded ETC. complexes, causing failure of mitochondrial bioenergetics. Moreover, defects in the axonal mitochondrial movement are also reported in patient-derived iPSCs (Mehta et al., 2021). The interaction of poly(GR)_80_ with mitochondrial ribosomal proteins is also reported to cause mitochondrial dysfunction. Further, the increased oxidative stress in such conditions induces DNA damage observed in the C9orf72 neurons (Lopez-Gonzalez et al., 2016). Onesto et al. report an increase in ROS levels in cells expressing mutated C9orf72 altering mitochondrial activity and might be the mechanism behind the cell death observed in ALS (Onesto et al., 2016). Moreover, the expression of redox genes was also observed to be impaired in ALS, where expression of the master regulator of redox genes, i.e., NRF2, is also reported to decrease in cells expressing DPRs (Jimenez-Villegas et al., 2022). On the other hand, the loss of C9orf72 functions is also linked with mitochondrial abnormalities, where C9orf72 controls assembly of complex-I, maintaining OXPHOS. The AIFM1/CHCHD4 mediates the import of C9orf72 to IMS from the cytosol, where C9orf72 inhibits the degradation of TIMMDC1, a chaperone involved in complex I formation (Wang et al., 2021). The C9orf72 DPRs are also shown to disrupt calcium homeostasis in neurons by affecting the ER-mitochondria association. Where C9orf72 mediated, GSK-3β activation disrupts ER-mitochondria (VAPB-PTPIP51) linkage (Gomez-Suaga et al., 2022). The involvement of C9orf72 in autophagy and lysosomal pathway is recently reported; further its depletion may result in autophagy defects observed in ALS pathology (Sullivan et al., 2016).

### 2.2 How mitochondrial dysfunction caused by mutant SOD1 contributes to neuronal death

Cu/Zn superoxide dismutase 1 (SOD1) was initially recognized by erythocuprein, which is essential in removing superoxide radicals generated during oxygen utilization in cellular systems (McCord and Fridovich, 1969). The SOD1 gene is positioned on chromosome 21q22.11 (human), having five exons encoding 154 extended amino acid chains forming monomers of the homodimeric SOD1 enzyme. Each monomer has a binding site for Cu^+^ and Zn^2+,^ which aids in the stabilization and catalytic activity of SOD1 (Siddique et al., 1991; Wei et al., 2017). SOD1 is predominantly present in the different regions of motor neurons, including dendrites, axons, and cell bodies (Pardo et al., 1995). Furthermore, it principally exists in cytosolic and mitochondrial regions where copper chaperone CCS helps import SOD1 to IMS of mitochondria (Field et al., 2003). SOD1 converts superoxide radicals generated in mitochondria (during OXPHOS) into H_2_O_2_ and molecular oxygen (Zelko et al., 2002). Finally, glutathione peroxidase transforms hydrogen peroxide to H_2_O, reducing the damage caused by superoxide radicals. Numerous mutations are identified in SOD1 that alter its metal binding properties and catalytic activity. The presence of such multiple mutations is also confirmed in fALS (Rosen, 1993; Hayward et al., 2002). The pathogenic variants of SOD1 affect mitochondrial functions and result in cellular death by apoptosis (Pasinelli et al., 2004).

The build-up of disease-causing SOD1 in mitochondria (spinal cord) is reported to affect its normal function (Liu et al., 2004). Further, mutated SOD1 interacts with mitochondria-associated Bcl2 protein, where this interaction causes structural changes in Bcl2 protein. The altered structure of Bcl2 (exposed BH3 domain) changes the mitochondrial structure, and ΔΨm results in *Cyt C* release*,* activating neuronal death by apoptosis (Pedrini et al., 2010). Furthermore, mutated SOD1 interaction with VDAC1 protein (at OMM) is also identified in mitochondria (spinal cord). This interaction alters the conductance of VDAC1, resulting in an increased cytosolic ADP/ATP ratio and a decrease in membrane potential (Israelson et al., 2010). The VDAC1 also provides a docking site for the hexokinase I (HK1), and this interaction helps to regulate ROS production and apoptosis (da-Silva et al., 2004). The depleted HK1 expression in the mouse spine compared to the brain is reported and may be a prime reason for selective damage of spinal cord mitochondria (Israelson et al., 2010). Recently Magri et al. identified SOD1 mutant and HK1 vying for VDAC1 and treatment of the N-terminal of HK1 in NSC34 cells protected mitochondria by inhibiting SOD1 (G93A) and VDAC1 interaction (Magri et al., 2016; Magri et al., 2021). The disturbed homeostasis of intracellular calcium is a key hallmark of ALS models, and mitochondria are also involved in calcium homeostasis. A study executed on the mutant SOD1 (G93A) expressing mice shows a decline in mitochondrial calcium uptake due to altered activity of mCU even though their expression appears normal (Damiano et al., 2006; Fuchs et al., 2013). The mutant SOD1 also modifies the mitochondrial dynamics, where it is found to induce levels of Drp1 and reduces the levels of OPA1, resulting in increased mitochondrial fragmentation (Raimondi et al., 2006; Ferri et al., 2010). In continuation, mitofusion activators in SOD1 (G93A) mice delay the ALS pathology by reducing mitochondria-related abnormalities (Dang et al., 2022). Furthermore, the mutated SOD1 interactions with the dynein protein is found to affect the retrograde transport of mitochondria (Zhang et al., 2007).

### 2.3 Disturbance of mitochondrial homeostasis: TDP-43 involvement

TDP-43 (43 kDa) is key disease-causing protein initially noted in ubiquitin-positive inclusions in different segments of CNS (neocortex, hippocampus, and spine) of ALS patients (Arai et al., 2006; Neumann et al., 2006). Multiple disease-linked TDP-43 mutants involvement in ALS pathogenicity are reported. TDP-43 gene is situated on chromosome 1p36.22 (human) (Sreedharan et al., 2008). TDP-43 shows structural similarity with heterogenous ribonucleoproteins (hnRNPs). It is principally found in nuclei and is involved in RNA processing (Buratti and Baralle, 2008; Freibaum et al., 2010). Conversely, cytoplasmic build-up of mutant TDP-43 is observed, affecting RNA processing of multiple proteins involved in neurodevelopment (Polymenidou et al., 2011; Tollervey et al., 2011). Both loss-and gain-of-function is evident in the TDP-43 associated pathomechanism (Sephton et al., 2010; Wils et al., 2010). TDP-43 is inherently inclined to form aggregates, where its C-terminal is crucial for spontaneous aggregate formation. Moreover, numerous mutations in this domain are identified, causing aggregate formation and inducing the associated pathogenicity (Johnson et al., 2009).

The disease-linked TDP-43 expressed in cells causes mitochondrial defects leading to cellular death. The TDP-43 depletes activity of complex I, resulting in ΔΨm loss (Lu et al., 2012). Interestingly, induced UCP2 levels were also observed in TDP-43 transfected cells showing a response against cellular injury. Recently, study has suggested that TDP-43 (full-length) may cause mitochondrial damage compared to truncated forms of TDP-43. TDP-43 interaction with mitochondrial mRNAs synthesizing complex I subunit (ND3/6), causing the impairment in its assembly and activity. The defective assembly of complex I further generate oxidative stress, inducing the formation of oligomers from truncated TDP-43 (Salvatori et al., 2018). Another study reports interacting partners of TDP-43, including VDAC1, PHB2, and Mfn2. TDP-43 with these interactors may modulate the autophagic clearance of mitochondria and its dynamics; however, the mechanistic details for this modulation are not entirely known (Davis et al., 2018). The increased levels of Fis1 and induced mitochondrial fragmentation are also reported in fibroblasts expressing mutant TDP-43 (Onesto et al., 2016).

The expression of TDP-43 (either truncated or full-length) was found to activate mitophagy with increased LC3 and decreased p62 levels. Furthermore, TDP-43 carboxy-terminal selectively reduces the VDAC1 levels (Hong et al., 2012). FUS and TDP-43 regulate the Parkin levels, and reduction in TDP-43 and FUS cause a decrease in Parkin levels (Lagier-Tourenne et al., 2012). ALS-associated disturbed ER-mitochondria connections also involve TDP-43, where activation of GSK-3β in TDP-43 dependent manner disrupts VAPB–PTPIP51 interaction. These disrupted connections also generate calcium disbalance leading to increased cytosolic calcium, further affecting other cellular processes, including axonal mitochondrial transport (Stoica et al., 2014). The anterograde mitochondrial movement requires kinesin-1, which interacts with mitochondria *via* Miro1 protein. Miro1 is a calcium-sensitive protein, and excess calcium impairs this transport pathway (Saxton and Hollenbeck, 2012). Moreover, TDP-43 and FUS control kinesin expression (Xiao et al., 2011; Colombrita et al., 2012). Following this, the altered mobility of mitochondria is observed in ALS disease models like *Drosophila* and patient-derived spinal motor neurons (Baldwin et al., 2016; Kreiter et al., 2018).

### 2.4 Altered function of FUS protein causes ALS-linked mitochondrial defects

FUS (RNA-interacting protein) is another ALS pathology protein, and mutation in this protein forms cytosol-localized aggregates in motor neurons affected in ALS (King et al., 2012). Initially, FUS has been identified as an oncogene causing liposarcoma and is alternatively termed as translocated in liposarcoma (TLS) (Crozat et al., 1993). FUS is one of FET family members, comprising other proteins, EWS and TAF15 (Tan and Manley, 2009). The gene encoding the FUS protein is situated in chromosome 16p11.2 (human), comprising 15 exons (Deng et al., 2014). The different domains in FUS protein include the SYGQ-rich region, RGG, RRM, ZnF domain, and nuclear localization and export signal sequence (Wang et al., 2015a). FUS is predominantly located at the nucleus, with its prominent role in DNA damage repair, gene expression, RNA processing, a regulator of cell division, and also implicated in stress response (Sama et al., 2014). The initial recognition of FUS as an oncoprotein led the research toward finding its involvement in cancer. In 2009, numerous mutations in FUS were reported to be linked with fALS. These mutations cause its mislocalization to the cytoplasm and forming inclusions responsible for the degeneration of motor neurons (Kwiatkowski et al., 2009; Vance et al., 2009). The NLS in the FUS protein is essential for its translocation to cellular nuclei by interacting with Karyopherinβ2, and mutation in this sequence results in its cytoplasmic retention (Bosco et al., 2010; Zhang and Chook, 2012).

The cytosolic accumulation of FUS also affects mitochondrial health, causing bioenergetic disbalance in a cell. The involvement of mitochondrial chaperone HSP60 in mitochondrial targeting of mutated FUS is reported by (Deng et al. 2015). Furthermore, the mutant FUS also binds with mitochondrial chaperones (Hsp70 and Hsp90), involved in transport of mitochondrial proteins from cytosol to mitochondria (Wang et al., 2015b). Mitochondria-localized FUS protein inhibits the ATP synthase formation by interacting with its catalytic subunit ATP5B. The FUS-dependent altered mitochondrial function activates UPR^mt^, and its extended activation results in neuronal death (Deng et al., 2018). Furthermore, the sequestration of mutant FUS protein with multiple mRNAs encoding, ETC. proteins is also reported. The aggregation of mRNA inhibits their translation, resulting in decreased levels of respiratory chain proteins affecting mitochondrial bioenergetics (Tsai et al., 2020). Nakaya and Maragkakis’s report also signify the defects in mitochondrial morphology in the presence of mutant FUS (R495X), which alters the expression of mitochondrial genes (Nakaya and Maragkakis, 2018). Mutant FUS may also be involved in the ER-mitochondria communication defects as it is identified to activate GSK-3β responsible for the VAPB–PTPIP51 defective interaction (Stoica et al., 2016). ALS-associated axonal cargo transport defects may involve FUS protein as it is recently recognized to interact with Syntaphilin (involved in mitochondrial tethering) and a mutated form of FUS alters this interaction causing mitochondrial abnormalities observed in ALS (Salam et al., 2021).

## 3 Unravelling the Alzheimer's disease associated mitochondrial impairments

**FIGURE 3:**
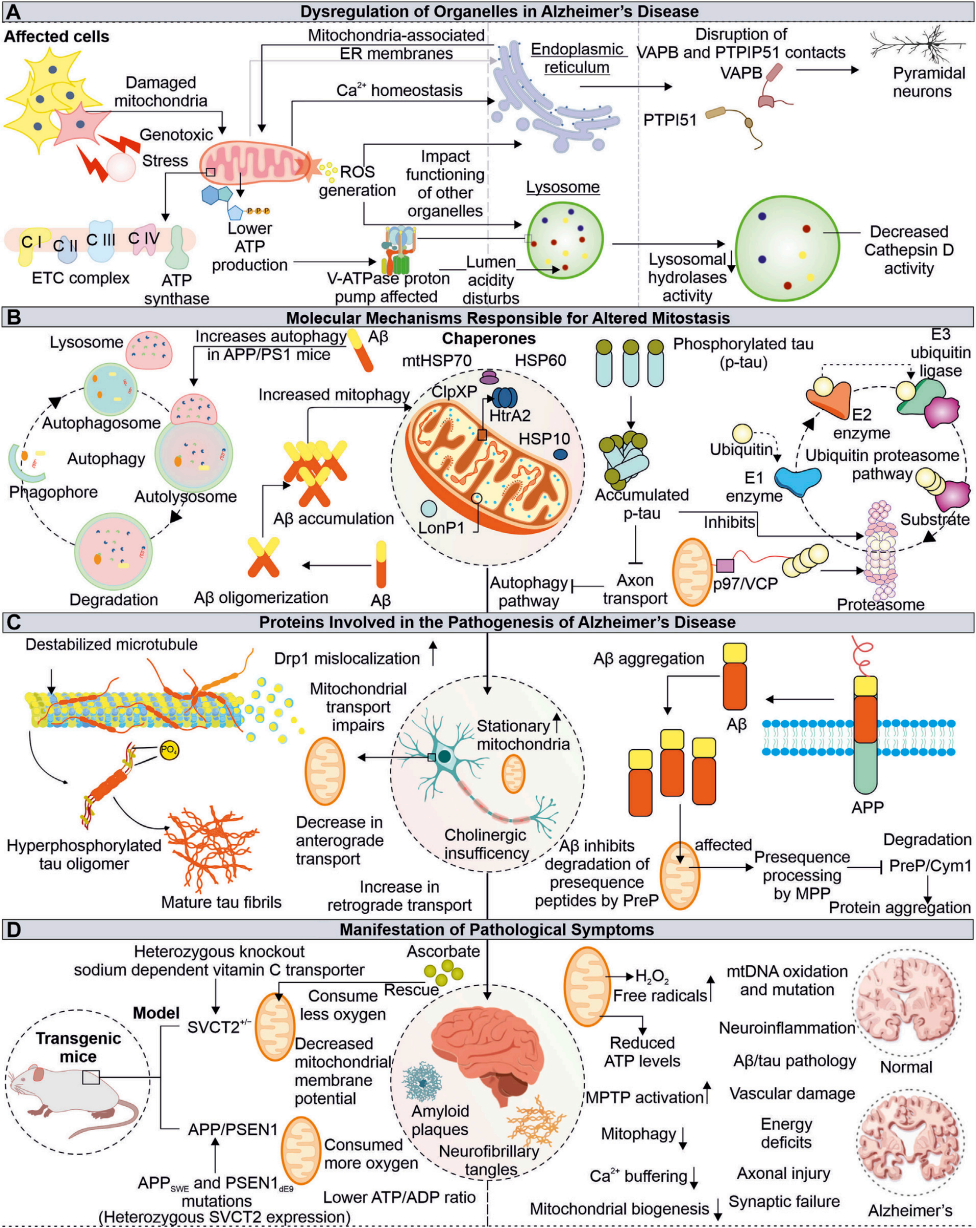
Impaired functions of Mitochondria in Alzheimer’s disease. Mitochondrial dysfunction due to pathogenic proteins from AD depletes the cellular energy, further affecting other organelles such as ER and lysosome **(A)**. The pathogenic Aβ and tau proteins are also identified to impair cellular quality control pathways, including autophagy and proteasome **(B)**. The tau and Aβ alter the normal mitochondrial functions and axonal transport **(C)**. The AD-associated physiological changes are linked with mitochondrial damage, where the misfolded Aβ and tau affect mitochondrial function at different levels **(D)**.

Alzheimer’s disease (AD) is complex neurological disorder generating atrophy of brain due to neuronal cells death resulting in dementia, majorly occurring at a later stage of life. The characteristic of AD is the intracytoplasmic neurofibrillary tangles (NFTs) made-up of proteins known as tau and extracellular senile plaques consisting of amyloid beta (Aβ). These pathogenic aggregates cause cellular defects, including mitochondrial damage, resulting in apoptosis-mediated neuronal death (Kumar et al., 2018). The toxic forms of tau and Aβ are extensively reported to adversely impact the neuronal cells of AD patients by impairing mitochondrial energy generation and antioxidation capacity (Figure 3).

### 3.1 Defective clearance of Aβ at mitochondria may affect import of mitochondrial proteins

In amyloidogenesis, step-wise splicing of β-amyloid precursor protein (APP) by β- and γ-secretases produce 37–43 amino acid-containing peptide known as Aβ (Haass et al., 2012). These Aβ peptides are amphipathic, forming amyloid fibers observed in AD patient brain sections (Soreghan et al., 1994; Hayden and Teplow, 2013). The animal models subjected to human Aβ42 oligomers have been shown to affect the number of synapses along with memory formation defects (Selkoe and Hardy, 2016). Furthermore, the binding of Aβ oligomers with metal ions like copper and iron can lead to a generation of ROS. The ROS further damages other cellular elements like lipids leading to neurotoxicity and cell death (Cheignon et al., 2018). The intracellular processing of APP at the site of mitochondria and ER contacts (MAM) leads to the production and internalization of Aβ to the mitochondria, where involvement of translocase of the outer membrane (TOM) is reported (Hansson Petersen et al., 2008; Area-Gomez et al., 2009). The Aβ targeted at mitochondria is degraded by hPreP, limiting its mitochondrial damage (Falkevall et al., 2006). However, during increased ROS levels, the inactivation of hPreP generates an Aβ build-up in mitochondria resulting in AD-associated death of neurons. Furthermore, reduced hPreP levels were reported in AD patient brains compared to control; thus, clearance of Aβ *via* hPreP can be explored as a therapeutic option (Alikhani et al., 2011; Teixeira et al., 2012).

Additionally, the occurrence of Aβ inhibits PreP resulting in accumulation of the presequence peptides usually processed by mitochondrial processing peptidase (MPP). The accumulation of presequence peptides inhibits MPP activity *via* a feedback mechanism, impairing mitochondrial presequence proteins' transport and resulting in mitochondrial impairments (Mossmann et al., 2014). The interaction of mitochondria located Aβ and Abeta-binding alcohol dehydrogenase (ABAD) alters ABAD function, leading to ROS generation, further causing mitochondrial dysfunction and death of neurons (Lustbader et al., 2004; Chen and Yan, 2007). Different inhibitors of ABAD-Aβ interaction are being tested for their possibility of reducing Aβ-linked neurotoxicity, and further development in this area can provide us with a viable therapeutic choice (Morsy and Trippier, 2019). Another approach for Alzheimer’s treatment is the clearance of Aβ from AD patient’s brains. Multiple drugs have been tried to achieve this objective with minimal success in clinical trials (Huang et al., 2020; Karran and De Strooper, 2022). Moreover, antibodies-based therapeutics for targeted clearance of Aβ were also explored, however recently FDA-approved aducanumab use for AD treatment remains controversial (Sevigny et al., 2016; Tampi et al., 2021). This further raise questions about correlation of Aβ pathology in AD-associated cognitive impairments, whether it is the cause or a feature of AD.

### 3.2 Tau associated mitochondrial defects and role of antioxidants in mitigating oxidative stress

Tau proteins are known to be associated with a microtubule, and AD-associated altered tau protein was also reported to cause mitochondrial defects. The hyperphosphorylated tau protein are identified in AD pathomechanism (Iqbal et al., 2010). The expression of tau protein was found to affect the cellular transport of multiple organelles, including mitochondria (Ebneth et al., 1998). Furthermore, the anterograde transport of mitochondria is affected in tau-overexpressed cells (Shahpasand et al., 2012). The mitochondrial dynamics defects, such as elongated mitochondria, are observed in human tau-expressed neuronal cells from *Drosophila* and mouse models. The impaired mitochondrial localization of Drp1 results in defective mitochondrial fission leading to elongation in mitochondria (DuBoff et al., 2012). Moreover, the aberrant interaction of Drp1 with hyperphosphorylated tau and Aβ leads to increased mitochondrial fission and fragmentation, damaging neuronal health (Manczak and Reddy, 2012). The combined toxic effects of Aβ and truncated tau (cleaved at Asp421) on mitochondria are observed in primary cortical neurons where the number of stationary mitochondria was increased along with loss of mitochondrial potential (Quintanilla et al., 2012).

The neuronal damage due to high oxidative stress in presence of AD-associated pathogenic proteins is another major phenomenon observed in AD patients (Uttara et al., 2009) (Lin and Beal, 2006). Further, the impaired antioxidant defense mechanism aggravates this neuronal damage, where numerous mitochondrial mechanisms, including its biogenesis, axonal transport, and dynamics, are also affected. The treatment of the mitochondria-specific antioxidant SS31 alleviates the above-mentioned effects (Calkins et al., 2011). The cellular system and mitochondria possess multiple antioxidant enzymes and ROS scavengers to regulate ROS levels (Mailloux, 2018). The decreased ROS scavengers, such as ascorbate (Vitamin C), accelerate the disease progression in transgenic APP/PSEN1 mice due to mitochondrial dysfunction. The altered levels of ascorbate can be used as a biological marker for the early detection of mitochondrial defects linked to disorders. Moreover, their decreased levels can be improved by dietary supplementation in elderly peoples prone to disease (Dixit et al., 2017).

## 4 How does mitochondrial dysfunction in AD lead to lysosomal and endoplasmic reticulum defects?

Mitochondrial communication with other cellular organelles is essential for maintaining homeostasis. The AD-associated mitochondrial impairments not only disturb cellular bioenergetics but also affects the functioning of other organelles, like lysosomes and ER. Although these organelles are compartmentalized and function independently in the cellular environment, they depend on mitochondria for energy. Different ER and lysosome defects associated with mitochondrial damage are reported in AD, which underlies the complex pathomechanism of neurodegeneration.

### 4.1 Alzheimer’s disease-related mitochondrial abnormalities and their impact on lysosome functions

The lysosomes are membrane-bound organelles engaged in degrading intracellular (autophagic pathway) and extracellular (endocytic pathway) components (Saftig and Klumperman, 2009). The acidic pH (4.5–5) of lysosome lumen drives its hydrolytic enzymes to carry out this activity. The vacuolar (H+) ATPase (or V-ATPase) drives proton into the lumen of lysosome using ATP and is primarily responsible for maintaining lysosome pH gradients (Mindell, 2012). The lysosomal acidification is impacted by V-ATPase dysfunction, which inhibits substrate clearance and causes a variety of illnesses, including neurodegenerative diseases (Song et al., 2020). Mitochondrial impairment causes a shortage of ATP, which can impact the V-ATPase activity; thus, lysosomal lumen pH maintenance is disturbed in disease conditions (Stepien et al., 2020). The initial belief is that the lysosome and mitochondria functions were independent but recently changed, with numerous studies showing their interdependency. Moreover, either lysosome or mitochondrial impaired function may affect others' functions, generating complex pathogenic conditions such as neuronal degeneration (Audano et al., 2018). The cortex and hippocampus tissue sections from APP/PS1 mice model show an accumulation of lysosomal marker lysosome-associated membrane protein 1 (LAMP1) along with enlarged lysosome structures around the aggregates of Aβ. This also represents impaired lysosomal degradation capacity. Further, the positive modulation of lysosomal enzyme cathepsin B (CatB) by PADK shows improved clearance of Aβ (Hwang et al., 2019). The decreased activity of CatB upon V-ATPase inhibition is also reported (Kubisch et al., 2014). This highlights the importance of V-ATPase in CatB-mediated clearance of Aβ and the connection of V-ATPase impairments due to mitochondria dysfunction observed in AD. Cathepsin D (CatD) is also reported to involve in clearing Aβ and tau, and its inhibition by Aβ42 is observed in the early onset of AD and can be utilized as a potential biomarker (Chai et al., 2019; Suire et al., 2020).

### 4.2 Interrupted endoplasmic reticulum-mitochondria communication in pathophysiology of Alzheimer’s disease

Mitochondria contacts with ER *via* MAMs are essential in multiple physiological functions of cells, including Ca^2+^ homeostasis, lipid synthesis, mitochondrial dynamics regulation, apoptosis, inflammation, and autophagy. The impaired crosstalk between these crucial organelles can be seen in multiple diseases, including neurodegeneration (Missiroli et al., 2018; Barazzuol et al., 2021). The enhanced interaction between Bcl-x_L_ and IP_3_R3 at MAM provides cellular protection during stress conditions, which is achieved by improved mitochondria-ER communication, calcium homeostasis, and cellular bioenergetics (Williams et al., 2016). Numerous crucial neural processes are modulated through the mitochondria and ER cross-talk, where multiple tethering proteins from both organelles crucially regulate this communication. One such connection is formed by the interaction of the surface proteins such as VAPB-PTPIP51 and is reported to be disrupted in degenerating neuronal conditions, like ALS, Parkinson, and FTD. Recently, the importance of these tethering proteins in autophagy regulation is reported, where loss of VAPB-PTPIP51 found to affect formation of autophagosome (Gomez-Suaga et al., 2017; Gómez-Suaga et al., 2019). Moreover, in post-mortem AD brain tissue analysis, the disrupted association between VAPB-PTPIP51 is observed in pyramidal neurons (temporal cortex) at the late stages of AD (Lau et al., 2020).

## 5 Pathogenic tau and Aβ affects cellular quality control pathways

The UPS and autophagy are crucial and essential cellular quality control pathways for maintaining homeostasis by clearance of misfolded and non-required proteins (Yang and Klionsky, 2010; Pohl and Dikic, 2019). These pathways also preserve the quality of cellular organelles, such as mitochondria. UPS is reported to modulate several mitochondrial pathways like its dynamics, mitophagy, and biogenesis (Ross et al., 2015; Bragoszewski et al., 2017). Moreover, the significance of cytosolic UPS in degrading OMM-localized proteins is also reported. Cdc48/VCP/p97 recruited at OMM assists in translocating them from mitochondria to cytosol (Fang et al., 2015). The inhibition of proteasome activity by Aβ is known further to accumulate Aβ and tau in aging-related disorders like AD (Oddo, 2008; Tseng et al., 2008). The impaired proteasome activity may damage mitochondrial health by altering its different quality control mechanisms, which further need to be studied and can be utilized as target AD-related therapeutics. The pathological interaction of p-tau and Aβ with Drp1 protein and Aβ-PINK1/Parkin affects the removal of damaged mitochondria from neurons. Furthermore, the age-linked accumulation of p-tau and Aβ causes depletion of multiple autophagy proteins (Reddy and Oliver, 2019). However, another report also suggests the induction of autophagy and mitophagy in cellular models treated with Aβ but not at sufficient levels to reduce Aβ. Moreover, reduced mitochondrial biogenesis and its increased fragmentation and mitophagy are observed in early pathogenic events in APP/PS1 mice (de la Cueva et al., 2022).

## 6 How do mitochondrial impairments and energy defects contribute to Parkinson's disease pathology?

**FIGURE 4:**
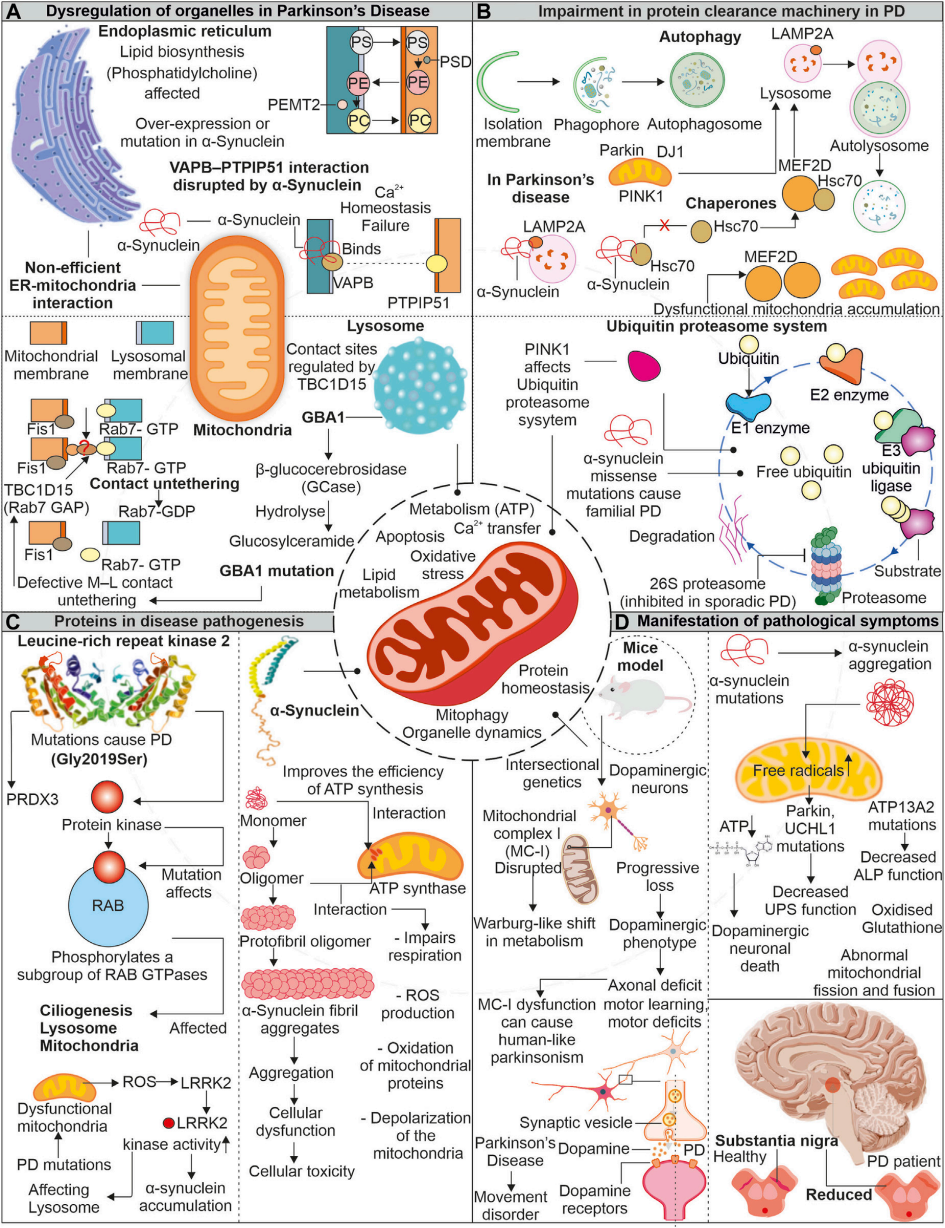
Mitochondrial impairment in Parkinson’s disease: Unraveling the connection. In the pathogenesis of the disease, mitochondria show dysfunctionality which affects other organelles functioning. Enzymes of lysosome like GBA1 is impaired due to mutation in PD, which affects the association of lysosome with mitochondria. Similarly, α-synuclein protein interferes with ER and mitochondrial association **(A)**. The impaired proteins involved in disease pathogenesis interact with autophagy and the UPS and interfere with the clearance of dysfunctional mitochondria **(B)**. Two proteins LRRK2 and α-synuclein, are reportedly intricate in disease pathogenesis. These proteins impair the functionality of mitochondria and increase oxidative stress **(C)**. Due to dysfunctional mitochondria, the dopamine-producing neurons are affected and later affect the brain region **(D)**.

Parkinson’s disease (PD) is often linked to imperfect aging and neuronal degeneration, with increasing occurrence globally. The disease symptoms include various motor function defects, frequently initiated with tremors, further progressing to loss of balance, muscle stiffness, and difficulty in coordination (Pringsheim et al., 2014; Kalia and Lang, 2015; Dorsey, 2018). Dopaminergic neurons exist in substantia nigra pars compacta (SNpc) are primarily affected in PD patients. Lewy bodies comprising aggregates of α-synuclein protein are identified in brain sections of PD patients (Figure 4). The continuous research on understanding the disease pathomechanism has improved our knowledge, and the involvement of multiple affected processes has been identified, such as increased oxidative stress, proteostasis defects, calcium dyshomeostasis, impaired axonal transport, and mitochondrial damage (Poewe et al., 2017). Research further highlights the mitochondrial impairments observed in PD patients with various mitochondria-associated proteins involved in PD pathology (Abou-Sleiman et al., 2006; Park et al., 2018).

### 6.1 Mutant proteins are implicated in the mitochondrial damage observed in Parkinson’s disease

The α-synuclein is a neuronal protein and acts as a chaperone promoting the assembly of SNARE-complex present in pre-synapse (Burre et al., 2010). The α-synuclein is known to affect dopaminergic neuronal mitochondria. Devi et al. reported increased α-synuclein accumulation, but not its lower levels in mitochondria generate bioenergetic defects by impairing the activity of complex I (Devi et al., 2008). Whereas recently, the unfolded monomeric form of α-synuclein is reported to interact and induce ATP synthase function. The increased levels of α-synuclein at pre-synapse are also suggested to positively modulate ATP synthase providing higher energy required for synaptic transmission mechanisms (Ludtmann et al., 2016). Moreover, the same group also showed the negative effect of oligomeric α-synuclein on ATP synthase, impairing mitochondrial respiration. Further, the depolarization of mitochondria in increased ROS levels causes opening of mitochondrial permeability transition pore (mPTP) (Ludtmann et al., 2018). Furthermore, the selectively impaired complex I activity is found to induce parkinsonism, where the altered release of dopamine is suggested as the prime mechanism for motor dysfunction (Gonzalez-Rodriguez et al., 2021). Intracellular oligomerization of α-synuclein is reported to occur at the membrane surface, where the mitochondrial lipid cardiolipin plays a prominent role in the oligomer formation. The hiPSC derived from α-synuclein (A53T) PD patients shows sequestration of cardiolipin in aggregates of lipid-protein complexes. Further, these aggregates were shown to cause mitochondrial defects leading to neuronal cell death (Choi et al., 2022). Moreover, the oligomers and fibrillar forms of α-synuclein can independently generate ROS in occurrence of metal ions (Deas et al., 2016). α-synuclein’s toxic oligomeric forms can harm cellular systems in various ways, such as damaging the cytoskeleton, hindering protein clearance, disrupting membranes, and causing mitochondrial depolarization (Roberts and Brown, 2015).

Leucine-rich repeat kinase 2 (LRRK2) is another protein from PD pathology, and multiple disease-causing mutations are identified in the LRRK2 gene. From several identified mutations, the G2019S mutation (kinase domain) is frequently observed in familial and sporadic PD (Gandhi et al., 2009). Furthermore, LRRK2 (G2019S) mutation was found to affect mitochondrial H_2_O_2_ homeostasis by altering the activity of mitochondria-localized peroxiredoxin-3 (PRDX3), a major H_2_O_2_ scavenger (Angeles et al., 2014). The increased mitochondrial fragmentation was observed in cells expressing LRRK2 (G2019S), which induces Drp1 phosphorylation at T595, increasing its activity (Su and Qi, 2013). Elevated oxidative stress in PD-affected neurons is a well-known fact. Further, this stress is reported to enhance LRRK2 kinase function, similar to LRRK2 (G2019S) (Rocha et al., 2022). The increased kinase LRRK2 activity is further found to induce phosphorylation of α-synuclein, leading to its enhanced accumulation observed in PD (Qing et al., 2009). Moreover, both proteins' co-localization is also reported in PD brain tissue sections and Lewy body inclusions (Guerreiro et al., 2013).

## 7 Parkinson's disease-associated mitochondrial defects contribute to the impairment of endoplasmic reticulum and lysosomal functions

Mitochondria continuously communicate with other cell organelles and are vital for numerous pathways functioning. Mitochondria interact with ER to regulate cytoplasmic calcium levels and lipid synthesis. Moreover, it is also essential for fission, fusion, and UPR^mt^. The disturbance in MAM contact sites is reported in numerous neurodegenerative disorders, including PD. Further, lysosomes and mitochondria are also associated *via* multiple membrane proteins regulating metabolite transfer and organelle dynamics. The disruption in these tethering proteins is also reported in PD (Wilson and Metzakopian, 2021; Cisneros et al., 2022).

### 7.1 Disrupted mitochondria- endoplasmic reticulum contacts in Parkinson’s disease

The mutated form of α-synuclein is identified to disturb the VAPB- PTPIP51 (ER-Mitochondria) association, further generating calcium dyshomeostasis and mitochondrial bioenergetic imbalances. The dopaminergic neurons with α-synuclein mutations also confirm the disturbed connection between VAPB-PTPIP51. However, this disrupted connection was not linked with GSK-3β altered activity and suggested that the binding of mutant α-synuclein with VAPB results in disrupted ER-mitochondrial association observed in PD (Paillusson et al., 2017). The ER-mitochondrial association is also essential for transporting multiple lipids required in mitochondrial functioning (Flis and Daum, 2013). Mitochondria and ER contact sites are necessary for synthesizing specific lipids like phosphatidylcholine (PC), where multiple intermediate steps require contact between these organelles (Vance, 1990; Flis and Daum, 2013). The phosphatidylserine (PS) generated in the presence of PS synthase 1/2 is further transported to mitochondria for transforming into phosphatidylethanolamine (PE), where PSD (a decarboxylase) role is necessary. The importance of PE at mitochondria for its morphology and function is reported. Again this PE from mitochondria can be transferred back into ER, where PEMT2 can convert it back to PC (Steenbergen et al., 2005; Joshi et al., 2012). Furthermore, PD models with the loss of Parkin/PINK1 functions show defects in sleep patterns and circadian cycles. Although the mitochondrial deformities were not predominantly observed in such conditions, the increased ER-mitochondrial associations were evident. This induced ER-mitochondrial association leads to the depletion of ER levels of phosphatidylserine, generating defects in the synthesis of neuropeptide-containing vesicles. Moreover, phosphatidylserine supplementation in disease models mitigates defective sleep patterns with improved synthesis of neuropeptide-containing vesicles (Valadas et al., 2018).

### 7.2 How do lysosomal abnormalities are associated with impaired mitochondria in Parkinson’s disease?

The mitochondrial contacts with lysosomes allow the organelles to communicate while maintaining cellular homeostasis. Various mitochondria-localized proteins control these organelle connections. The RAB7 protein from the lysosome assists in tethering with mitochondria, where Fis1 recruited TBC1D15 disrupts these connections by converting RAB7 from GTP (active) to GDP (inactive) form (Wong et al., 2018; Wong et al., 2019). The lysosomal functions are essential for removing the misfolded proteins, and its impaired activity is observed in PD. This lysosomal dysfunction is linked with impaired mitochondria. The increase in oxidized dopamine due to mitochondria-linked oxidative stress led to a decrease in lysosomal glucocerebrosidase activity, which further accumulates α-synuclein (Burbulla et al., 2017). The lysosomal and autophagic defects are observed in PD patient-derived neurons with a mutation in lysosomal enzyme β-glucocerebrosidase. Further, these neurons also represent calcium dyshomeostasis with increased cytosol calcium levels (Schondorf et al., 2014). Restoring β-glucocerebrosidase enzyme activity improves the lysosome-linked clearance of α-synuclein (Mazzulli et al., 2016). The mitochondrial respiration and axonal transport are also affected in PD patient-derived dopaminergic neurons having the β-glucocerebrosidase mutation. The observed mitochondrial defects were linked with altered mitochondria-lysosome contacts, where prolonged tethering due to decreased activity of TBC1D15 is identified (Kim et al., 2021). The altered activity of lysosome-linked ATP13A2 affects lysosomes at multiple levels; further, it impairs autophagy, resulting in α-synuclein aggregation (Zhang et al., 2022).

## 8 Autophagy and proteasomal defects associated with Parkinson disease

Chaperone-mediated autophagy (CMA), a selective type of autophagy, aids in removal of soluble cytosolic protein having a KFERQ-like pentapeptide sequence. Cytosolic Hsc70 and co-chaperones such as the HSC70-HSP90 organizing protein (HOP), HSP40, and CHIP identify this sequence on target proteins, further facilitating their translocation across the lysosomal membrane *via* LAMP2A receptor and resulting in protein breakdown (Kaushik and Cuervo, 2018). The native α-synuclein acts as a substrate for CMA-dependent degradation (Vogiatzi et al., 2008). Moreover, the mutant α-synuclein (A53T and A30P) was observed to inhibit the CMA by interacting with LAMP2A, further affecting the degradation of other CMA substrates and contributing to increased cellular stress (Cuervo et al., 2004). The transcriptional controller MEF2 was shown to have a crucial role in neuronal survival (Mao et al., 1999). Their suppression causes the death of neurons while increasing their activity guards against harmful cellular stress (Tang et al., 2005; Smith et al., 2006). The suppressed CMA in the presence of α-synuclein affects the interaction of MEF2D with Hsc70, resulting in its accumulation and loss of function, causing neuronal death (Yang et al., 2009). The neuronal accumulation of multiple PD-associated misfolded proteins positive for ubiquitin also suggests the impairment of another quality control pathway, i.e., UPS. Furthermore, PD-linked Lewy bodies were reported positive for 20S proteasome and α-synuclein protein. Moreover, the impaired proteolytic activity due to α-synuclein oligomers is also shown (Lindersson et al., 2004). PINK1 mutants from PD pathology generates mitochondrial bioenergetic defects, which further affect other cellular energy-dependent processes including UPS-mediated degradation (Liu et al., 2009).

## 9 Mitochondria: A promising therapeutic target for neurodegenerative disorders

The decline in mitochondrial function during aging and associated disorders like neurodegeneration has received much attention, and there are extensive efforts underway to develop pharmacological treatments that can restore the potential and integrity of these crucial organelles. A large number of pharmacological modulators, both natural and synthetic, are being studied for their ability to reduce mitochondrial stress by targeting different pathways, including mitochondrial OXPHOS, ROS homeostasis, and metabolic processes. Furthermore, several other pathways, such as mitochondrial biogenesis, dynamics, and degradation, are also considered in developing therapeutics against mitochondria-associated disorders.

The depleted energy production by mitochondria due to various cellular stresses such as increased ROS levels and calcium dyshomeostasis, significantly affects energy-intensive cells like neurons. Scientists have explored various molecules that could potentially improve the functioning of the, ETC. For example, riboflavin and idebenone enhance the transfer of electrons, while others, such as thiamine and dichloroacetate, increase the availability of, ETC., substrates (Bernsen et al., 1993; Barshop et al., 2004; Haefeli et al., 2011; Mermigkis et al., 2013). The promising effects of idebenone are noted in multiple studies on individuals with Alzheimer’s disease (Senin et al., 1992). In addition, researchers have studied various natural compounds for their ability to regulate mitochondrial oxidative stress. For example, saponins derived from *Panax japonicus* and *Panax notoginseng* show neuroprotective effects by reducing mitochondrial damage through the induction of antioxidant responses (Zhou et al., 2018; Wan et al., 2020). Besides this, several mitochondrial antioxidants, including MitoQ, Mitotempo, and Mito apocynin, protects mitochondria from oxidative damage (Hu and Li, 2016; Brenza et al., 2017; Young and Franklin, 2019).

Another approach to address energy deficiency involves increasing the number of mitochondria in cells by targeting transcription factors participating in formation of new mitochondria, such as PGC-1α. Pioglitazone, a type of thiazolidinedione, has been shown to have protective effects in several neurological diseases (Masciopinto et al., 2012; Morato et al., 2013). Pioglitazone induces mitochondrial biogenesis and mtDNA copy number by targeting transcription factors such as PGC-1α (Bogacka et al., 2005). Furthermore, various studies show the protective effects of directly or indirectly activating mitochondrial biogenesis with compounds such as bezafibrate, resveratrol, and AICAR (Komen and Thorburn, 2014). It is evident that mitochondrial dynamics are altered in various neurodegenerative diseases, and different pharmacological interventions that can modulate the proteins involved in this process are investigated. Echinacoside (ECH) treatment shows neuroprotective effects by inducing mitochondrial fusion *via* increased transcription of Mfn2 (Zeng et al., 2021). Treatment with liquiritigenin, a flavonoid, has been found to induce mitochondrial fusion and protects against Aβ cytotoxicity (Liu et al., 2011; Jo et al., 2016).

Additionally, inhibiting the mitochondrial fission pathway has been found to have therapeutic benefits. For example, the use of mitochondrial division inhibitor-1 (Mdivi-1) decreases mitochondrial fragmentation (inhibit Drp1) in cells treated with Aβ, as well as maintains ΔΨm and prevents *Cyto C* release (Cassidy-Stone et al., 2008; Xie et al., 2014). Various modulators of mitophagy that have shown beneficial effects by removing damaged or altered mitochondria are identified. Urolithin A induces autophagic removal of altered mitochondria and extended lifespan in *C. elegans* (Ryu et al., 2016). Spermidine treatment was found to induce both the mitophagy as well as biogenesis of mitochondria in aged mice heart cells (Messerer et al., 2023). Metformin, a drug used in treating T2D, has been found to enhance mitochondrial function by restoring, ETC., proteins and promoting mitophagy (de Maranon et al., 2022).

## 10 Conclusion and future outlook

Mitochondria are crucial cell organelles well-recognized for their involvement in ATP production, and they also take part in numerous cellular signaling and pathways. Primarily, these include homeostasis of calcium, synthesis of biomolecules, and regulation of cell death by apoptosis. The impairment in such mitochondrial functions is detected in numerous pathological conditions, including metabolic, neurodevelopmental, neurodegeneration, and cancer. Moreover, mitochondrial dysfunction at early stages of neurodegeneration is prominently observed in ALS, AD, and PD. The neurons affected in neurodegenerative disease are significantly dependent on mitochondria for numerous functions, for instance, neurogenesis, neuronal development, neurotransmission, and neuronal repair pathways. Different neurodegenerative disease-causing proteins are identified to affect the mitochondria resulting in bioenergetic defects and increased ROS production. The ALS-linked C9orf72, FUS, SOD1, TDP-43, and PD-linked α-synuclein oligomers were found to affect mitochondrial, ETC., protein function by either influencing their activity, expression of subunits or directly impairing their assembly. The OXPHOS-impaired mitochondria act as source of ROS, inducing oxidative stress in neurons. Moreover, the inadequate antioxidant response further worsens the damage. The age-linked depletion in ROS homeostasis and other quality control pathways further aggravates the mitochondria damage leading to cell death by apoptosis.

Mitochondria possess different quality control mechanisms to circumvent such situations, including chaperones and proteases. UPR^mt^ activated upon increase in mitochondrial stress communicates with the nucleus for induced expression of mitostasis proteins. The dynamics and autophagy of mitochondria also regulate its health by either diluting the mitochondrial damage (fusion) or degrading dysfunctional mitochondria (fission and mitophagy). The pathogenic misfolded proteins from neurodegenerative diseases are known to interfere with these mitostasis pathways. The AD (Aβ, tau), ALS (SOD1), and PD (LRRK2) pathogenic proteins interact and alter fission proteins inducing aberrant mitochondrial fragmentation. These misfolded proteins can disturb mitochondrial contact with ER and lysosomes, resulting in impairment of calcium homeostasis and autophagy. The increased cytosolic calcium due to dysfunctional ER-mitochondria connection is also linked with defective mitochondrial transport. The negative impact of disease-misfolded proteins on cellular protein quality control (PQC) machinery hampers mitochondrial quality control, intensifying the damage to multiple mitochondria-dependent cellular processes. Despite the existence of many sophisticated methods for understanding the pathogenesis of disorders, no effective treatments are currently available for mitochondrial-associated diseases. Therapeutic approaches for mitochondrial diseases are presently limited to enhancing electron transfer in OXPHOS, using antioxidants, and stimulating mitochondrial biogenesis, among others. Furthermore, mitochondrial gene therapy, mitochondrial protein replacement therapy, mitochondrial transplantation, and nanoparticle drug delivery can be potential ways to advance mitochondrial disease therapeutics.

Developing therapeutic agents and strategies for neurodegeneration is a challenging task, given the insufficient understanding of complexity of their initiation and pathogenesis. Mitochondria can serve as ideal candidates for the connecting link between multiple complex degenerating disorders. Understanding role of mitochondria, mechanism of their dyshomeostasis, and its effect on cellular health and survival can provide us insight into the basic mechanistic aspects of neurodegeneration. The investigation of mitochondrial dysfunction in network with other associated organelles in neurodegeneration allows us to target mitochondria specifically or in combination with other organelles. The current therapeutic landscape includes many targets for neuronal degeneration, such as cholinergic, glutamatergic, and dopaminergic systems, but the clinical results are less than satisfactory. The multiple mitochondrial molecules and pathways discussed in this review can be investigated for their diagnostic and therapeutic potential and advancing the understanding of neurodegeneration.

## Glossary

AD: Alzheimer’s disease
AICAR: 5-Aminoimidazole-4-carboxamide ribonucleotide
AIFM1: apoptosis inducing factor mitochondrial 1
APP: Amyloid precursor protein
ATP5A1: ATP synthase F1 subunit alpha
Aβ: Amyloid beta
Bcl2: B-cell leukemia/lymphoma two protein
C9orf72: chromosome nine open reading frame 72
CatB: Cathepsin B
CatD: Cathepsin D
CCS: copper chaperone for superoxide dismutase
CHCHD4: Coiled-coil-helix-coiled-coil-helix domain-containing protein 4
Clp: Caseinolytic proteins
ClpP: Caseinolytic protease P
CM: Cristae membrane
CMA: Chaperone-mediated autophagy
CNS: Central nervous system
COX17p: Cytochrome c oxidase copper chaperone
Cyt *C*: Cytochrome *C*
**DNAJA3**DnaJ homolog subfamily A member 3: mitochondrial
Dnm1: Dynamin-related protein DNM1
DPRs: Dipeptide repeats
Drp1: Dynamin-related protein 1
ER: Endoplasmic reticulum
ERAL1: ERA-like protein 1
ERRα: Estrogen-related receptor α
ETC: Electron transport chain
EWS: Ewing Sarcoma
fALS: familial ALS
Fis1: Mitochondrial fission one protein
FTD: Frontotemporal dementia
fTLD: Frontotemporal lobar degeneration
FUS: Fused in sarcoma
GSK3β: Glycogen synthase kinase-3 beta
HIF1α: Hypoxia inducible factor 1 subunit alpha
HK1: hexokinase I
hnRNPs: Heterogenous ribonucleoproteins
HOP: HSC70-HSP90 organizing protein
hPreP: Human presequence protease
HRE: hexanucleotide (GGGGCC) repeats expansion
HSC20: Heat shock cognate protein 20
HtrA2: High temperature requirement protein A2
i-AAA: Intermembrane space AAA
IBM: Inner boundary membrane
IMM: Inner mitochondrial membrane
IMS: Intermembrane space
**IP**_**3**_ **R3**: Type 3 inositol 1,4,5-tris-phosphate receptors
iPSCs: Induced pluripotent stem cells
LAMP1: lysosome-associated membrane protein 1
LC3: Light chain 3
LHON: Leber hereditary optic neuropathy
LonP: Lon protease
MAM: mitochondrial-associated ER membrane
MBR: Metal-binding region
MCU: mitochondrial calcium uniporter
MERRF: Myoclonic epilepsy with ragged red fibers
Mfn1: Mitofusin-1
Mfn2: mitofusin two
MICU1: Mitochondrial calcium uptake one
mtDNA: mitochondria DNA
ND3: NADH-ubiquinone oxidoreductase chain 3
NFTs: Neurofibrillary tangles
NF-κB: Nuclear factor kappa B
NHK1: HK1-N-terminal based peptide
NLS: Nuclear localization signal
NRF1: Nuclear respiratory factor 1
NRF2: Nuclear factor erythroid 2-Related Factor 2
OMA1: Overlapping With The M-AAA Protease 1 Homolog
OMM: Outer mitochondrial membrane
OPA1: optic atrophy protein 1
OPTN: Optineurin
OXPHOS: oxidative phosphorylation
PADK: Z-Phe-Ala-diazomethylketone
PC: Phosphatidylcholine
PD: Parkinson’s disease
PE: Phosphatidylethanolamine
PEMT2: Phosphatidylethanolamine N-methyltransferase
PFN1: Profilin one
PGC-1α: Peroxisome proliferator-activated receptor-gamma coactivator 1 alpha
PHB2: Prohibitin-2
PINK1: PTEN-induced putative kinase 1
poly (GA): Glycine-Alanine
poly(GP): Glycine-Proline
poly(GR): Glycine-Arginine
poly(PA): Proline-Alanine
poly (PR): Proline-Arginine
PS: Phosphatidylserine
PSD: Phosphatidylserine decarboxylase
PTPIP51: Protein tyrosine phosphatase interacting protein-51
RGG: Arg–Gly–Gly-rich motif
ROS: Reactive oxygen species
RRM: RNA-recognition motif
SNpc: substantia nigra pars compacta
SOD1: Cu/Zn superoxide dismutase one
SQSTM1: Sequestosome one
SYGQ-rich region: (serine tyrosine glycine glutamine)-rich region
T2D: type 2 diabetes
TAF15: TATA-box binding protein associated factor 15
TBC1D15: TBC1 domain family member 15
TDP-43: TAR DNA-binding protein 43
TFAM: transcription factor A Mitochondrial
TFB1M: Transcription Factor B1 Mitochondrial
TFB2M: Transcription factor B2 Mitochondrial
TIMMDC1: Translocase of inner mitochondrial membrane domain containing one
TLS: Translocated in liposarcoma
TOM: Translocase of the outer membrane
UBQLN2: Ubiquilin two
UCP2: Uncoupling protein two
**UPR** ^**mt**^: Mitochondrial unfolded protein response
UV: Ultraviolet
VAPB: Vesicle-associated membrane-protein-associated protein B
V-ATPase: Vacuolar (H+) ATPase
VCP: Valosin-containing protein
VDAC1: Voltage-dependent anion-selective channel protein 1
WTL: Wild-type like
ZnF: Zinc-finger motif
